# LncRNA TP73‐AS1 enhances the malignant properties of pancreatic ductal adenocarcinoma by increasing MMP14 expression through miRNA ‐200a sponging

**DOI:** 10.1111/jcmm.16425

**Published:** 2021-03-08

**Authors:** Haiyan Miao, Jingjing Lu, Yibing Guo, Hongquan Qiu, Yu Zhang, Xihao Yao, Xiaohong Li, Yuhua Lu

**Affiliations:** ^1^ Research Center of Clinical Medical and Department of General Surgery Affiliated Hospital of Nantong University Nantong China; ^2^ Department of General Surgery The Sixth People's Hospital of Nantong Nantong China; ^3^ Visitor scholar of Wake Forest Institute for Regenerative Medicine Wake Forest University School of Medicine Winston‐Salem NC USA

**Keywords:** ceRNA, miR‐200a, mmp14, pancreatic ductal adenocarcinoma, TP73‐AS1

## Abstract

Pancreatic ductal adenocarcinoma (PDAC) is an invasive and aggressive cancer that remains a major threat to human health across the globe. Despite advances in cancer treatments and diagnosis, the prognosis of PDAC patients remains poor. New and more effective PDAC therapies are therefore urgently required. In this study, we identified a novel host factor, namely the LncRNA TP73‐AS1, as overexpressed in PDAC tissues compared to adjacent healthy tissue samples. The overexpression of TP‐73‐AS1 was found to correlate with both PDAC stage and lymph node metastasis. To reveal its role in PDCA, we targeted TP73‐AS1 using LnRNA inhibitors in a range of pancreatic cancer (PC) cell lines. We found that the inhibition of TP73‐AS1 led to a loss of MMP14 expression in PC cells and significantly inhibited their migratory and invasive capacity. No effects of TP73‐AS1 on cell survival or proliferation were observed. Mechanistically, we found that TP73‐AS1 suppressed the expression of the known oncogenic miR‐200a. Taken together, these data highlight the prognostic potential of TP73‐AS1 for PC patients and highlight it as a potential anti‐PDAC therapeutic target.

## 1 INTRODUCTION

Pancreatic cancer (PC) is an important and aggressive malignancy with an undesirable prognosis.^1^ Although surgery offers therapeutic potential for PC, post‐operative mortality rates remain high, with the overall survival (OS) rates reported to be as low as 8%.^2^ Moreover, PC patients are typically diagnosed at advanced stages due to the limitations in diagnostic techniques and atypical symptoms.^3^ Despite improvements in the treatment of many cancer types in the dawn of precision medicine and technological advances, improvements in PC have been limited. New therapeutic strategies and diagnostic biomarkers based on a deeper understanding of the key PC drivers are therefore urgently required.

LncRNAs are dysregulated in a range of cancers.^4^ LncRNA TP73 (TP73‐AS1) is a member of TP53 family and a known post‐transcriptional regulator of TP73 expression.^5^, ^6^ TP73‐AS1 influences tumorigenesis through its activity as a competitive endogenous RNA (ceRNA).^7^ That suppresses miR‐200a to enhance hepatocellular carcinoma (HCC) cell proliferation, mediated through the HMGB1/RAGE axis.^8^ TP73‐AS1 also promotes colorectal cancer (CRC) development through its ability to regulate TGFα signalling, an effect achieved through the sponging of miR‐194.^9^ TP73‐AS1 also targets miR‐449a in lung cancer cells to enhance the expression of zeste homolog 2 (EZH2) and promote cancer development.^10^ A role of TP73‐AS1 in the development of PDAC has, however, not been fully defined.

MicroRNAs (miRNAs) are ~23 RNA molecules that are non‐coding and regulate gene expression.^11^ miR‐200a in a range of important human malignancies including breast, colon, cervical, ovarian, colon and liver cancer.^12^ MiR‐200a is induced by a range of physiological stimuli, including IL‐9, that enhances miR‐200a/β‐Catenin expression and subsequent cancer cell metastasis.^13^ Low levels of miR‐93 and miR‐200a expression in cancer cells are also associated with a loss of differentiation in PDAC,^14^ while its overexpression enhances chemo‐resistance and MT1‐MMP expression in PC cells.^15^, ^16^ MiR‐200a can inhibit PC metastasis through its ability to suppress DEK.^17^ In our previous studies, we confirmed that miR‐200a is suppressed in PC stem cells^18^ but the involvement of TP73‐AS1 was not reported.

Here, we identify TP73‐AS1 as overexpressed in PDAC tissues compared to adjacent healthy tissue samples. The overexpression of TP‐73‐AS1 was correlated with both PDAC stage and lymph node metastasis. To reveal its role in PDCA, we targeted TP73‐AS1 using LnRNA inhibitors in PC cells. The inhibition of TP73‐AS1 led to a loss of MMP14 expression in PC cells and significantly inhibited their metastatic capacity. TP73‐AS1 was further found to up‐regulate MMP14 expression through its suppression of miR‐200a. These data provide new insight into the pro‐oncogenic effects of the TP73‐AS1/miR‐200a axis during PC development.

## 2 METHODS

### 2.1 Cell culture and samples

The PC cells PANC‐1, miacapa‐2, BxPC‐3 and non‐PC cells HPDE6C7 were purchased from the Shanghai Cell Bank. HPDE6C7, PANC‐1 and BxPC‐3 cells were cultured in DMEM plus 10% FBS (Invitrogen Biotech Co, Ltd) and 1% pen/strep at 5% CO_2_ and 37°C, and Miacapa‐2 cells were grown in DMEM supplemented with 5% horse serum. We obtained non‐tumour and PC tissues from patients who received Pancreaticoduodenectomy in the Affiliated Hospital of Nantong University from 2013 to 2017. The study was approved by our ethics committee, and informed consent was provided by all participants. All tissue samples were snap‐frozen and stored prior to use.

### 2.2 Tissue microarray

The immunohistochemical staining rate was classified as 0 (negative), 1 (1%‐25% positive tumour cells), 2 (26%‐50%), 3 (51%‐75%) and 4 (76%‐100%). Staining intensity was classified as 0 (absence of stained cells), 1 (weak staining), 2 (moderate staining) and 3 (strong staining). The immunohistochemistry (IHC) score was calculated by multiplying the staining rate and intensity. The final expression scores of TP73 were calculated with the value of per cent positivity score multiplied by staining intensity score, which ranged from 0 to 4. The degree of protein staining was quantified using a two‐level grading system, and when the sample was scored ≥2 point, we defined it as high expression, otherwise low expression or negative.

### 2.3 CCK‐8 assay

For cell proliferation assessments, transfected Panc‐1 and Miacapa‐2 cells (3 × 10^4^/mL) were seeded into 96 well for the indicated times and treated with CCK‐8 reagent (10 µL) at 37°C for 2 hours. Absorbances were read at 450 nm on a microplate reader.

### 2.4 Transwell migration assay

Digest and collect each group of pancreatic cancer cells after transfection. Resuspend and dilute the cells with RPMI‐1640 basal medium to adjust the cell density to (1‐10)×10^5^/mL. Place the Transwell chamber in a 24‐well plate, add 600 µL 20% foetal bovine serum RPMI‐1640 complete medium to the lower chamber, add 100 µL cell suspension (about 5 × 10^4^ cells) to the upper chamber. Cells were fixed after 48 hours of incubation with methanol and stained with 0.1% crystal violet. The number of cells invading through the membrane was counted under a light microscope (three random fields per well).

### 2.5 Transwell invasion assay

Place the Transwell chamber in a 24‐well plate in advance, spread a layer of Matrigel in the upper chamber and incubate it in a cell culture incubator for 5‐6 hours. After the Matrigel has solidified, the steps are the same as the transwell migration assay.

### 2.6 Apoptosis analysis

For apoptosis assays, cells were transfected for 48 hours and resuspended in 1 × binging buffer. Cells were then stained with 7‐AAD, PE, 7‐AAD+ PE and assessed by flow cytometry. Blank (unstained groups) were included as controls. For the assessment of cell cycle progression, transfected PC cells were fixed in 70% ethanol at 4°C overnight, RNAase A treated and stained with PI. Cells were assessed by flow cytometry.

### 2.7 Dual‐luciferase reporter assays

HEK293T cells (1.5 × 10^4^/well) were co‐transfected with TP73AS1‐WT1420, TP73AS1‐WT3830, TP73AS1‐MT1420 and TP73AS1‐MT3830, with miR‐200a mimics or mimic NC for 48 hours. Dual‐Luciferase Reporter Assays were then performed as per the manufacturer's protocols (Promega).

### 2.8 RT‐qPCR

RNA was extracted from PC cells or tumour tissues via TRIzol lysis, and cDNA was synthesized using Rayscript Kits. RT‐qPCRs were performed using a qPCR PreMix Kit on an ABI7500 RT‐PCR System. U6 was assessed as a control to which miR‐200a expression was normalized. TP73‐AS1 and MMPs were normalized to GAPDH. The 2^‐ΔΔCT^ method was used to quantify relative gene expression. The primers of the molecules used in the article are listed in Table 1. Primer sequences were synthesized by Generay Co Ltd.

**TABLE 1:** Primers used for RT‐qPCR

Primer	Forward (5′–3′)	Reverse (5′ –3′)
lncRNA TP73‐AS1	GGACCCATCAGACTCACGACA	CAAAGGGCTCAGACAAACAGG
GAPDH	GGACCAATACGACCAAATCCG	AGCCACATCGCTCAGACAC
miR‐200a‐3p	ACACTCCAGCTGGGTAACACTG	CTCAACTGGTGTCGTGGAGTCG
	TCTGGTAAC	GCAATTCAGTTGAGACATCG
U6	CTCGCTTCGGCAGCACA	AACGCTTCACGAATTTGCGT
MMP‐1	CTCTGGAGTAATGTCACACCTCT	TGTTGGTCCACCTTTCATCTTC
MMP‐2	ATTCCGCTTCCAGGGCACA	GGTCTCAGGGCAGAAGCCATAC
MMP‐14	CTGCCTGCGTCCATCAACACT	GTTCCAGGGACGCCTCATCA
MMP‐9	CCCTGGTCCTGGTGCTCCTG	CTGCCTGTCGGTGAGATTGGTTC

### 2.9 RNA FISH

A total of 58 frozen tissue samples harvested from PC patients were sectioned (~5 µm thick), washed in PBS and fixed in 3.7% formaldehyde for 10 minutes. Sections were then washed in 10% formamide and labelled with hybridization solution containing 10% formamide and 10% dextran sulphate (w/v) at 37°C overnight. Slides were then washed in 10% formamide and DAPI stained. TP73‐AS1 was labelled with rhodamine probes. Sections were rinsed in 2 × sodium citrate buffer and assessed by confocal microscopy.

### 2.10 Western blot

For WB analysis, PC cells were lysed in RIPA buffer and resolved by SDS‐PAGE electrophoresis. Proteins were then transferred to PVDF membranes and blocked with 5% 2092 M XUE ET AL pure milk for 1 hour and labelled with primary antibodies to MMP14 and GAPDH (1:1000, CST, USA) at 4°C overnight. Membranes were then labelled with the appropriate conjugated secondary antibodies.

### 2.11 Cell transfection

For in vitro experiments, TP73‐AS1 NC/si‐TP73‐AS1 and miR‐200a inhibitor nc/miR‐200a inhibitors were transfected using riboFECTCP transfection reagent (RiboBio Biotech). TP73‐AS1 was silenced using lentiviral vectors (LV‐si‐TP73‐AS1, target sequence ^14^:5′‐GCACCATTCCTGAGAAATA‐3′). All lentiviral vectors expressed GFP. Efficiencies were confirmed by RT‐qPCR and GFP imaging via fluorescent microscopy.

### 2.12 Metastasis models in nude mice

To assess the in vivo roles of TP73‐AS1, BALB/c nude mice (4‐6 weeks old, n = 12) from the laboratory animal centre of Nantong University were divided into 4 groups (n = 3). Stably transfected Panc‐1 and Miacapa‐2 cells (2 × 10^7^ cells/mL in PBS) were injected into the abdominal cavity of BALB/c nude mice. Mice were sacrificed at week 5, and the number of tumours was assessed. Excised tissues were H & E stained to assess MMP14 expression. The tumour tissues were stored at −80℃ for use.

### 2.13 Immunohistochemistry

Nude mouse tumour tissues were fixed in 4% formaldehyde, paraffin embedded and sectioned. Sections were probed with Anti‐MMP14 antibodies (Abcam) and counterstained with haematoxylin. Sections were then dehydrated, mounted and imaged.

### 2.14 Statistics

All data are shown as mean ± standard deviation. Data were analysed using SPSS 20.0 software (IBM SPSS Statistics). Student's

*t* test, one‐way analysis of variance or a – chi‐square test was used to nalyse the differences between two or more groups. *P* <.05 was deemed a statistical difference.

## 3 RESULTS

### 3.1 TP73‐AS1 is overexpressed in PC cells

**FIGURE 1:**
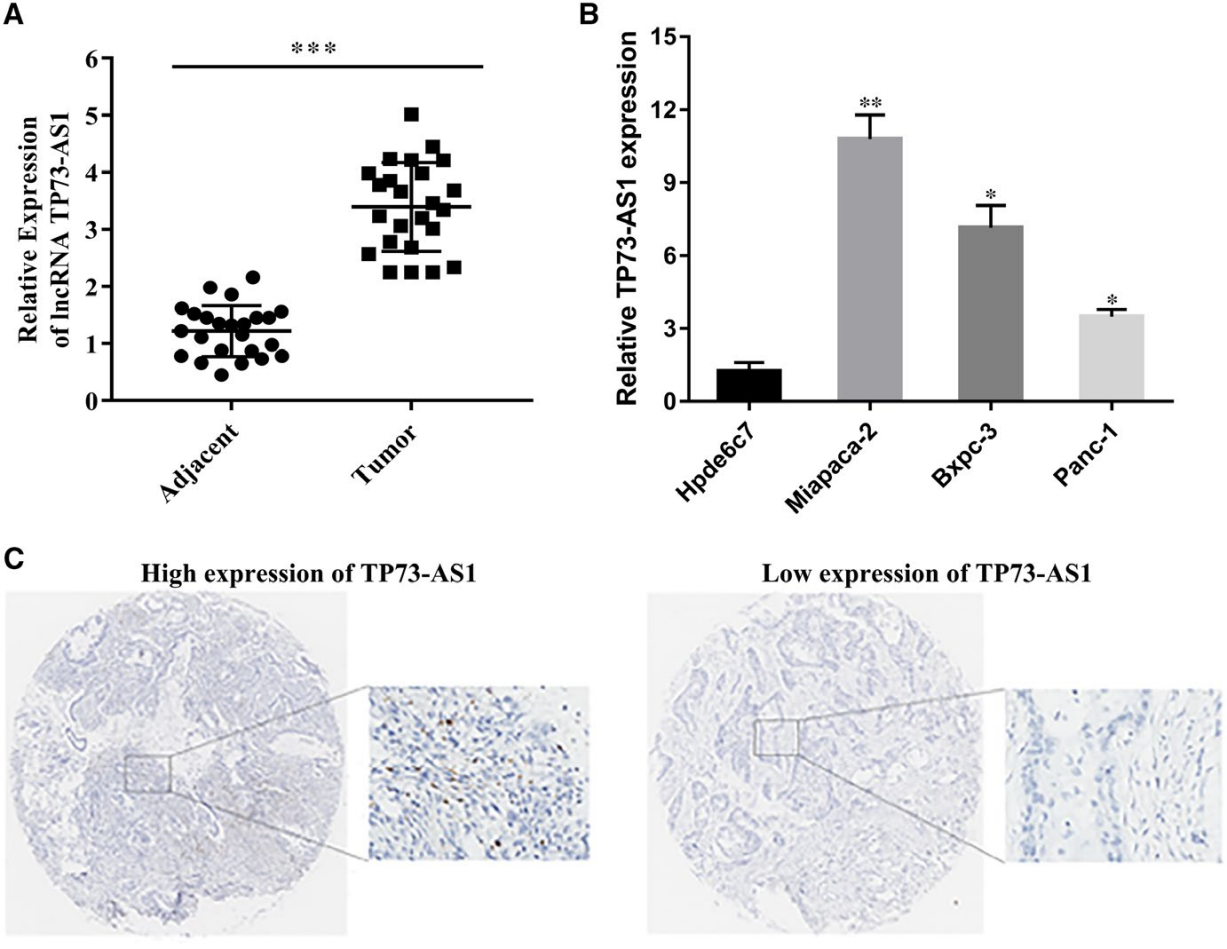
TP73‐AS1 was overexpressed in PC. A, TP73‐AS1 expression in adjacent normal pancreatic tissues and PC tissues analysed by RT‐qPCR. Levels were normalized to GAPDH. The 2^‐ΔΔCT^ method was used to quantify relative gene expression. B, TP73‐AS1 levels in HPDE6‐C7, PANC‐1, miacapa‐2 and BxPC‐3 cell lines measured by RT‐qPCR. C, TP73‐AS1 expression in the 58 PC tissues (*P <.05, **P <.01, ***P <.001)

To explore the role of TP73‐AS1 during PC progression, we analysed its expression in paracancerous samples (n = 24). The results demonstrated that TP73‐AS1 is overexpressed in cancer tissues (Figure 1A), PC cells (Panc‐1, miacapa‐2, Bxpc‐3) and normal pancreatic epithelial cell (Figure 1B). These data highlighted TP73‐AS1 as a potential oncogene. For further verification, the expression of TP73‐AS1 was detected in a tumour microarray containing 58 cancer tissues from patients (Figure 1C). According to the basic clinical and pathological parameters of tumours, patients with pancreatic cancer are grouped according to age, sex, tumour TNM stage and lymph node metastasis. The results showed that the high expression of TP73‐AS1 in pancreatic cancer tissue was related to tumour TNM stage (*P* =.001) and lymph node metastasis (*P* =.018), and the difference was statistically significant. There was no significant difference with the patient's gender, age (*P* >.05) (Table 2).

**TABLE 2:** Characteristics of 58 patients with PC

Characteristics	Number of patients	Expression of TP73‐AS1		χ^2^ value	*P* value
		Low	High		
Age (years)					
<60	39	20	19	0.078	.78
≥60	19	9	10		
Gender					
Male	32	17	15	0.279	.597
Female	26	12	14		
TMN stage					
I‐II	32	23	9	13.663	.001**
III‐IV	26	6	20		
Lymph node metastasis					
Negative	31	20	11	5.613	.018*
Positive	27	9	18		

* *P* < .05.

** *P* < .01.

### 3.2 TP73‐AS1 enhances PC metastasis by inhibiting MMP14 expression

**FIGURE 2:**
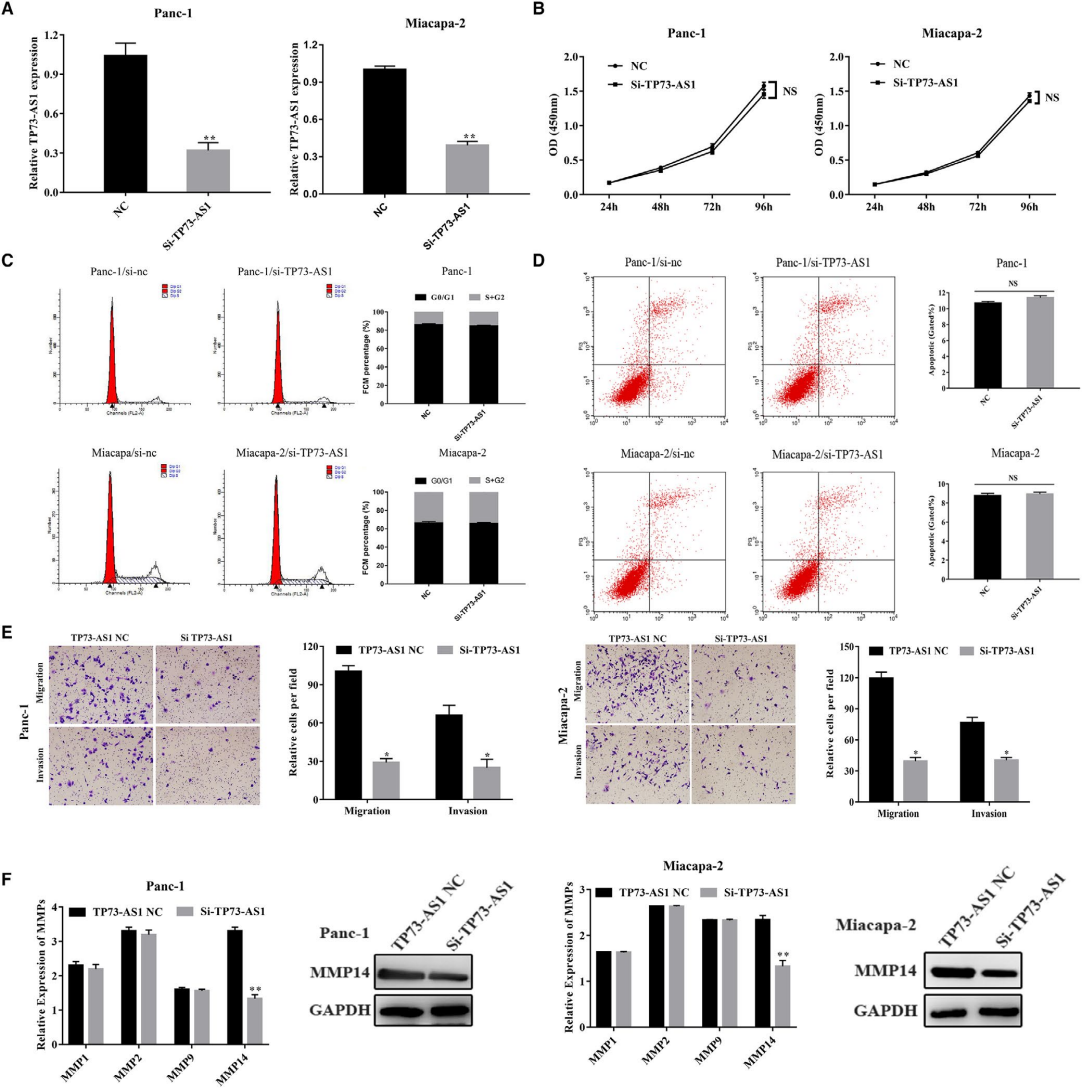
Down‐regulation of TP73‐AS1 inhibited the migration and invasion of PC cells. A, Inhibitory efficiency of si‐TP‐AS1. B, TP73‐AS1 knock‐down had no effect on cell viability of PC cells. C, TP73‐AS1 knock‐down had no effect on cell cycle progression of PC cells. D, TP73‐AS1 knock‐down had no effect on apoptosis of PC cells. E, TP73‐AS1 knock‐down inhibited migration and invasion of PC cells. F, MMPs expressions were assessed via RT‐qPCR and WB. N = 3, mean ± SD. (NS: No significance, *P <.05, **P <.01, ***P <.001)

To evaluate its role in PC, TP73‐AS1 was silenced through si‐TP73‐AS1 transfection. RT‐qPCR analysis confirmed effective TP73‐AS1 silencing (Figure 2A). Following si‐TP73‐AS1 transfection, the invasion, migration, proliferation, cell cycle and cell apoptosis of PC cells were assessed by transwell, cck‐8 and flow cytometry assays, respectively. We found that the invasion and migration of si‐TP73‐AS1 groups were lower than the NC group, but PC cell survival, cell cycle progression and cell apoptosis were unaffected (Figure 2B‐E). MMPs degrade the extracellular matrix and are up‐regulated in many tumours. We detected MMP expression in Panc‐1 and Miacapa‐2 cells transfected with si‐TP73‐AS1 and NC by RT‐qPCR. We found that MMP14 was down‐regulated in si‐TP73‐AS1 groups vs NC groups (Figure 2F). These data indicated that TP73‐AS1 influenced the metastatic phenotypes of PC cells by inhibiting MMP14 expression.

### 3.3 TP73‐targets miR‐200a to regulate MMP14 expression

**FIGURE 3:**
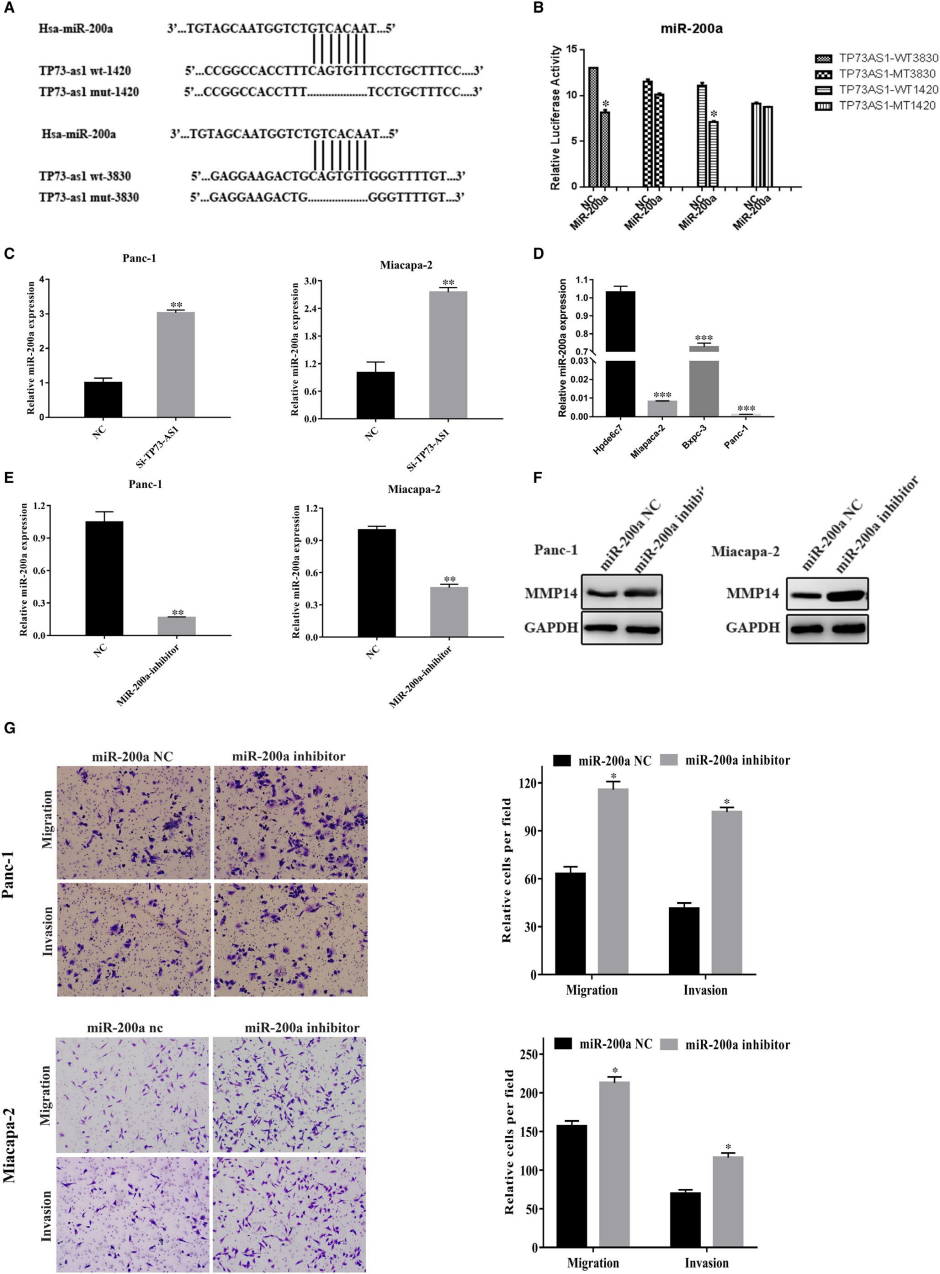
TP73‐AS1 targeted miR‐200a in PC cells. A, The binding site of TP73‐AS1 and miR‐200a. B, Detection of the activity of dual‐luciferase reporter gene. C, MiR‐200a levels following TP73‐AS1 silencing. D, MiR‐200a expression in PC cells and HPDE6C7. E, The effect of pancreatic cancer cells transfected with miR‐200a inhibitors or NC. F, Migratory and invasiveness of cells treated with miR‐200a inhibitors. G, Expression of MMP14 in miR‐200a inhibitor transfected cells. (*P <.05, **P <.01, ***P <.001)

**FIGURE 4:**
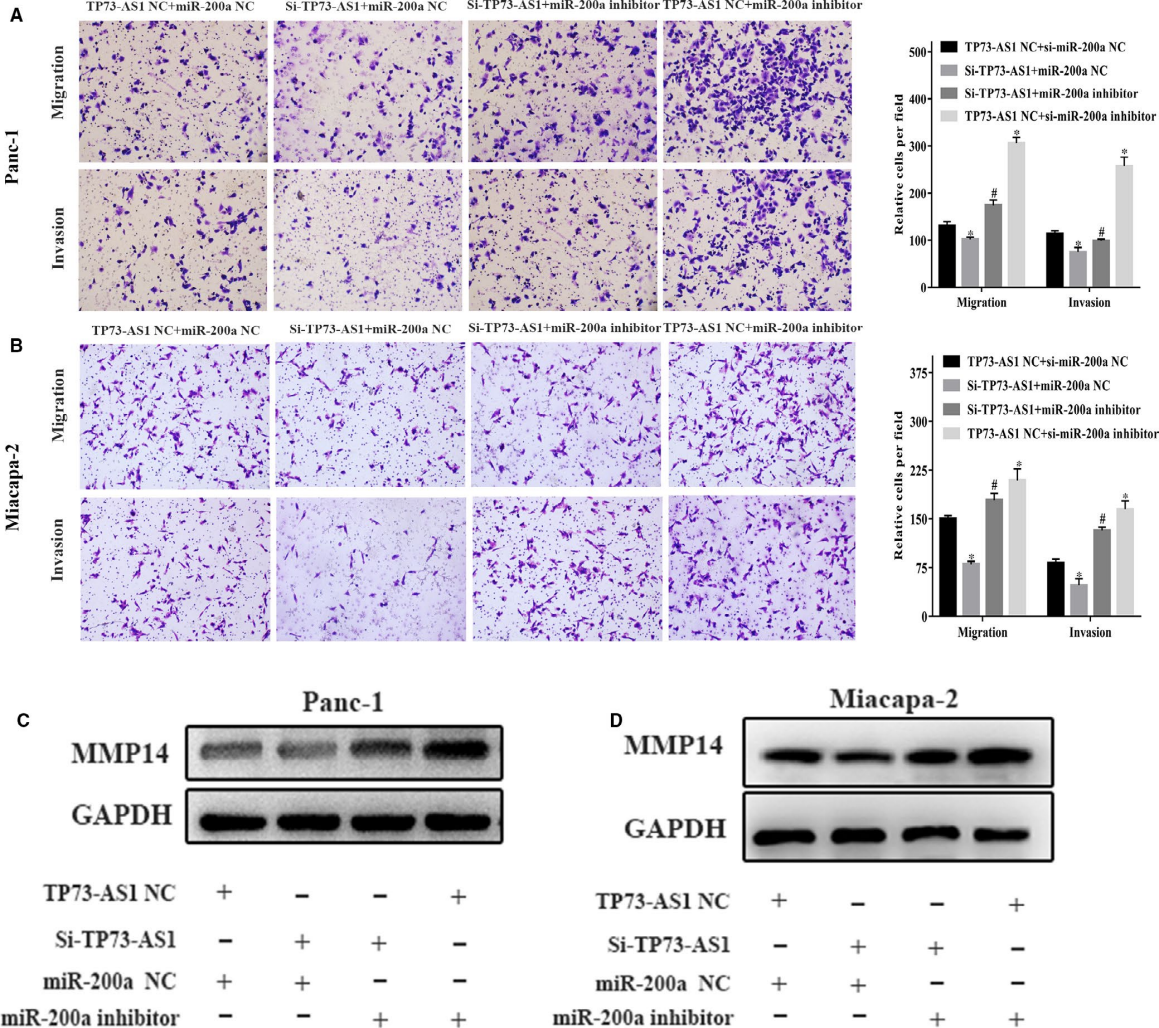
MiR‐200a reversed TP73‐AS1 functions. A and B, Transwell assays of invasion and migration in PC cells after co‐transfection of TP73‐AS1 nc/si‐TP73‐AS1 and miR‐200a or nc/miR‐200a inhibitors. C and D, Westernblot assays the expression of MMP14 in Panc‐1 and Miapaca‐2 co‐transfected with TP73‐AS1 or nc/si‐TP73‐AS1 and miR‐200a inhibitors or nc/miR‐200a inhibitors. (*P <.05, **P <.01, ***P <.001)

LncRNAs affect tumour progression through miRNA targeting. We therefore assessed TP73‐AS1 as a potential miR‐200a target through TargetScan (http://starbase.sysu.edu.cn/starbase2/browseNcRNA.php). We therefore chose miR‐200a for subsequent research (Figure 3A). Dual‐luciferase reporter assays were performed to confirm this interaction. The data showed that miR‐200a mimics suppressed the activity of TP73‐AS1‐wt reporters in HEK293T cells but had no effects on TP73‐AS1‐mut cells (Figure 3B). In addition, TP73‐AS1 silencing significantly increased miR‐200a expression in Panc‐1 and Miacapa‐2 cells (Figure 3C). MiR‐200a was suppressed to higher levels in PC compared to non‐PC Hpde6c7 cells (Figure 3D). Following transfection with miR‐200a inhibitors, miR‐200a expression significantly declined (Figure 3E), while MMP14 protein was significantly overexpressed (Figure 3F). The invasion and migratory ability of Panc‐1 and Miacapa‐2 cells was also significantly higher than that NC inhibitor‐group by transwell assays (Figure 3G). Furthermore, the co‐transfection of miR‐200a inhibitors into PC cells expressing si‐TP73‐AS1 could rescue the loss of PC cell invasion and migration (Figure 4A‐B). Western blot analysis further confirmed that the expression of MMP14 was rescued by the co‐transfection of miR‐200a inhibitors into PC cells transfected with si‐TP73‐AS1 (Figure 4C‐D). These data suggest that TP73‐AS1 suppresses PC cell MMP14 expression through miR‐200a targeting.

### 3.4 TP73‐AS1 regulates PC metastasis in vivo

**FIGURE 5:**
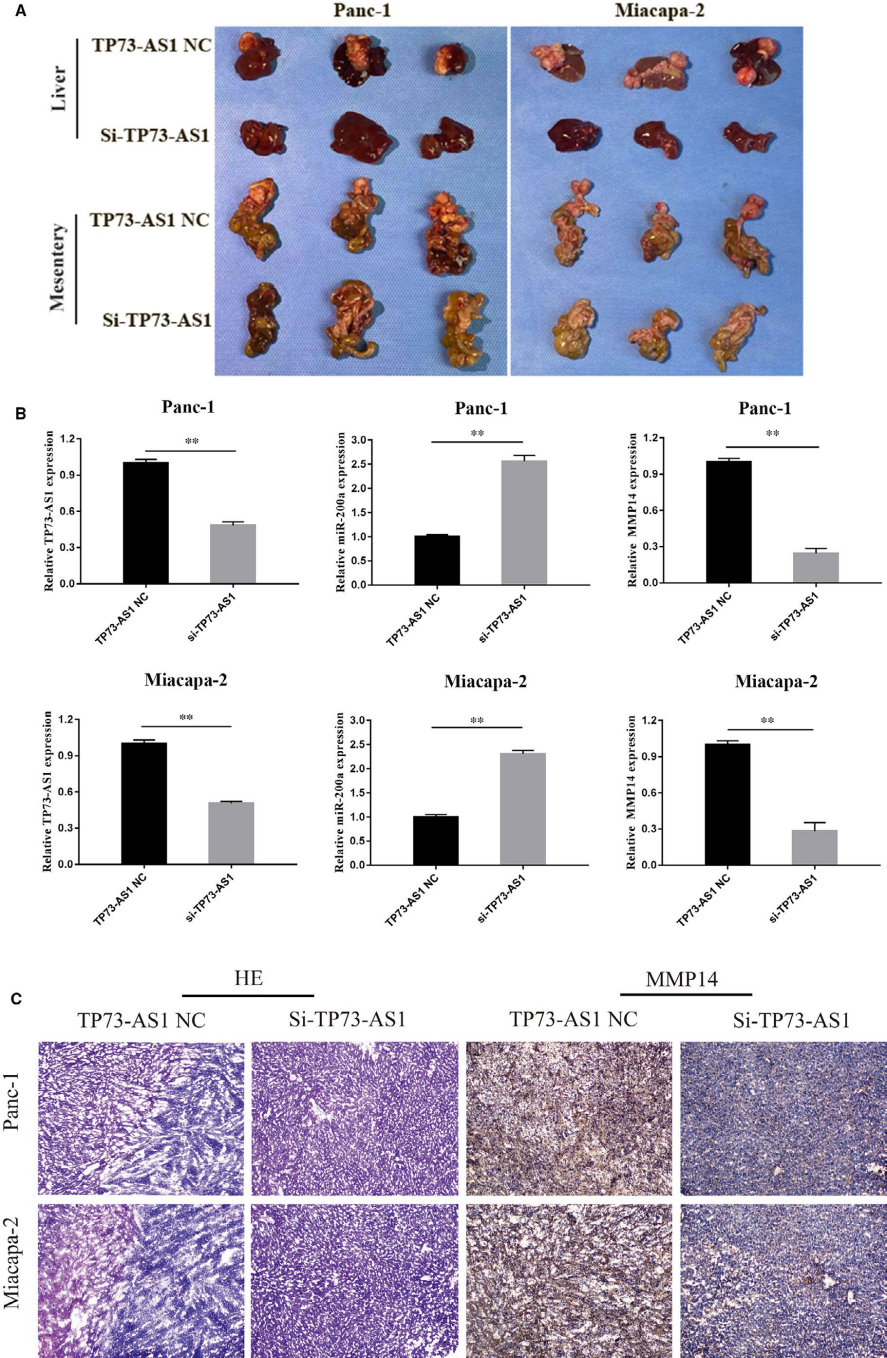
Low expression of TP73‐AS1 suppressed the liver metastasis of PC cells in vivo. A, Images of excised livers and mesenteries of BALB/c nude mice treated with Si‐TP73‐AS1 or TP73‐AS1‐NC. B, We detected the TP73‐AS1, miRNA‐200a and MMP14 expression in liver metastasis by RT‐qPCR (**P <.01). (C) Representative images of haematoxylin and eosin (HE) stained sections and MMP14 immunohistochemistry showing livers with or without tumours (magnification ×40)

**FIGURE 6:**
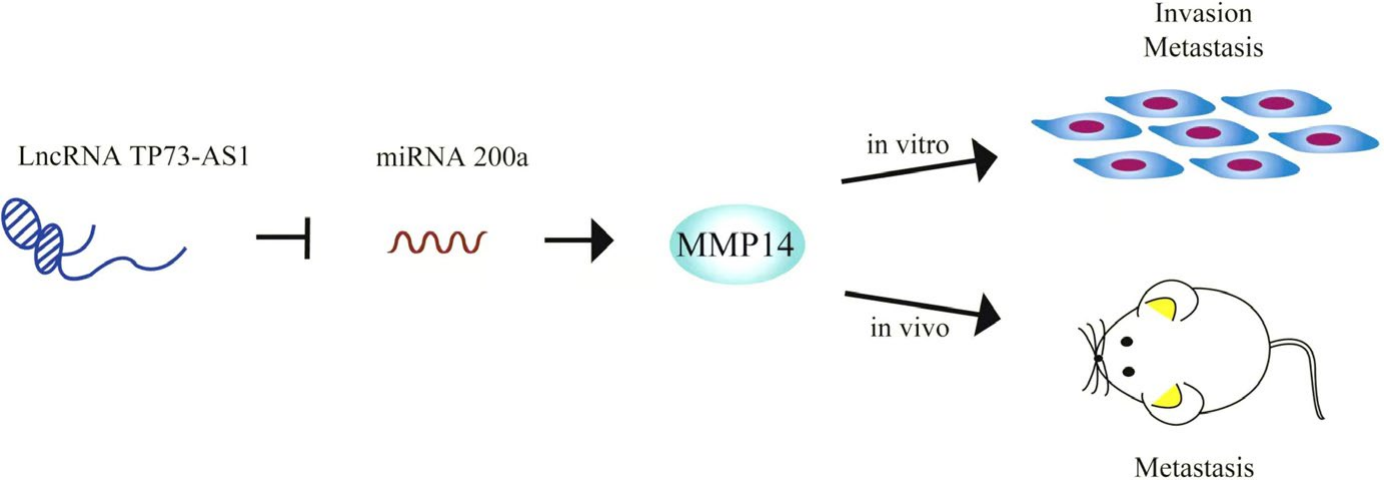
Schematic diagram of molecular mechanism of lncRNA TP73‐AS1 and miR‐200a in PC

To investigate the effects of TP73‐AS1 in vivo, xenograft mice bearing LV‐si‐TP73‐AS1‐transfected Panc‐1 and Miacapa‐2 cells were established as in vivo tumour models. The images of excised livers and mesenteries of BALB/c nude mice treated with Si‐TP73‐AS1 or TP73‐AS1‐NC were showed in Figure 5A. Tumour metastasis in livers and mesenteries were suppressed in TP73‐AS1 low expression groups (Panc‐1 si‐group 1/3 metastases in liver and 1/3 metastases in Mesentery, Miacapa‐2 si‐group 0/3 metastases in liver and 0/3 metastases in Mesentery) compared to the control group (Panc‐1 nc group 3/3 metastases in liver and 3/3 metastases in Mesentery, Miacapa‐2 nc group 3/3 metastases in liver and 3/3 metastases in Mesentery ). Moreover, we detected the expression levels of TP73‐AS1, miRNA‐200a and MMP14 in liver metastasis by RT‐qPCR in Figure 5B. The results showed that the expression of MMP‐14 was lower in LV‐si‐TP73‐AS1‐transfected group, while the expression of miR‐200a was higher in LV‐si‐TP73‐AS1‐transfected group, compared with nc group in liver metastasis (Figure 5B). Furthermore, immunohistochemical (IHC) staining showed that the expression of the MMP14 was lower in LV‐si‐TP73‐AS1 group compared to LV‐control group (Figure 5C). All these results indicated that LV‐si‐TP73‐AS1 could inhibit the metastasis capability of PC cells in vivo. Taken together, our data indicated that lncRNA TP73‐AS1 can combine with miR‐200a to promote the metastasis of PC cells in vivo and in vitro (Figure 6).

## 4 DISCUSSION

Pancreatic cancer (PC) is a malignant tumour that despite advances in surgical resection, radiotherapy, chemotherapy and immunotherapy, has one of the lowest 5‐year survival rates. Due to the lack of early diagnostics, PC is often diagnosed as late and advanced stages leading to a dismal prognosis for afflicted patients.^19^ By 2030, PC is predicted to be the 2nd leading cause of US cancer‐associated deaths.^2^ PC has a similarly poor prognosis in European countries with close to 1 000 000 thought to have died from PC.^20^ New and effective diagnostics are therefore urgently required. In this study, we found that lnc‐TP73‐AS1 is highly expressed in PC patients and cells and is associated with lymph node metastasis and tumour staging. In vivo and in vitro studies characterized the ability of TP73‐AS1 to form a ceRNA network that regulates MMP14 expression, a key driver of PC metastasis, an effect mediated through the direct targeting of miR‐200a. These data highlight TP73‐AS1 as an important mediator of PC progression, and a novel PC diagnostic and therapeutic target.

A range of lncRNAs have been shown to promote tumour progression. LncRNAs can act as suppressors of oncogenes or can promote metastatic phenotypes.^21^ LncRNAs interact with mRNAs or miRNAs to regulate transcription, chromosomal interactions, transcription factor binding, chromatin cyclization, gene methylation, transcription factor recruitment and chromatin modifications.^22^, ^23^, ^24^ The LncRNA TP73‐AS1 is a ceRNA of miRNA‐103 that modulates hepatoma cell proliferation and promotes gastric cancer (GC) cell cisplatin resistance.^25^, ^26^ Given this knowledge, this study investigated the contribution of TP73‐AS1 to PC development.

In 24 PC patients, TP73‐AS1 was found to be significantly overexpressed. Similar data were observed in PC cell lines compared to non‐PC epithelial cells. Through analysing the clinical data of 58 PC patients, the elevated expression of TP73‐AS1 positively correlated with TNM stage and lymph node metastasis. TP73‐AS1 silencing inhibited the metastatic phenotypes of PC cells, highlighting its role as an oncogene during PDAC development.

MMPs are zinc‐containing endopeptidases that degrade the extracellular matrix during tumour progression.^27^ MMPs are highly expressed in PDAC where they promote cancer cell invasion. Arginine deprivation is known to inhibit PC cell metastasis and EMT through its ability to dampen the expression of Slug, Snail, Twist and MMP1/9.^28^ Stress‐induced phosphoprotein 1 promotes PC progression through its ability to activate FAK/AKT/MMP signalling.^29^ MMP‐2 and TGF‐RI in the circulating tumour cells of PC patients are closely associated with disease severity.^30^ Tumour cell MMP3 expression is a prognostic for poor survival in pancreatic, pulmonary and mammary carcinoma.^31^ We therefore confirmed whether TP73‐AS1 influenced the invasive ability of PC cells through its action on MMPs. TP73‐AS1 silencing significantly suppressed MMP14 expression in Panc‐1 and Miapaca‐2 cells, but had no effects on other MMPs.

Knowledge of the roles of lncRNAs and their regulatory mechanisms are increasing. Specific lncRNAs act as molecular sponges to sequester miRNAs and regulate their function. Bioinformatics analysis highlighted potential binding sites in lncRNA TP73‐AS1 and miR‐200a, inferring it acts as a molecular sponge for miR‐200a. Dual‐luciferase reporter assays confirmed that TP73‐AS1 binds to miR‐200a, which was identified as poorly expressed in PC cells. Consistent with these findings, miR‐200a enhanced the metastatic phenotypes of PC cells and increased MMP14 expression.

To further verify TP73‐AS1 binding to miR‐200a, silencing experiments were performed. The invasion and migration ability and MMP14 expression of PC cells decreased after TP73‐AS1 silencing. In cells silenced for TP73‐AS1 and treated with miR‐200a inhibitors, the effects of TP73‐AS1 on metastatic invasion, migration and the inhibition of MMP14 expression were eliminated. TP73‐AS1 was also found to negatively correlate with miR‐200a expression in PC cells. In vivo experiments confirmed the loss of metastatic phenotypes of PC cells following TP73‐AS1 silencing. This suggested that TP73‐AS1 promotes the metastasis of PC cells in vivo through its effects on miR‐200a and subsequent MMP14 expression. The effects of TP73‐AS1 on the metastatic processes of PC cells can therefore be explained through a ceRNA mechanism.

In summary, we have identified TP73‐AS1 was an important oncogene in the metastasis of PC through its ability to regulate MMP14 and miR‐200a expression in PC cells. As a result, the TP73‐AS1/miR200a/MMP14 axis represented a promising target for PC therapeutics.

## CONFLICT OF INTEREST

The authors confirm that there are no conflicts of interest.

## AUTHOR CONTRIBUTION

**haiyan miao:** Data curation (equal); Methodology (equal); Software (equal); Writing‐original draft (equal); Writing‐review & editing (equal). **Jingjing Lu:** Data curation (equal); Methodology (equal); Project administration (equal); Resources (equal); Software (equal). **Yibing Guo:** Methodology (equal); Resources (equal); Software (equal). **Hongquan Qiu:** Data curation (equal); Methodology (equal); Supervision (equal); Writing‐original draft (equal). **Yu Zhang:** Methodology (equal); Software (equal); Writing‐original draft (equal); Writing‐review & editing (equal). **Xihao Yao:** Data curation (equal); Writing‐original draft (equal). **Xiaohong Li:** Data curation (equal); Methodology (equal); Resources (equal); Software (equal). **Yuhua Lu:** Funding acquisition (equal); Methodology (equal); Resources (equal).

## Data Availability

All data which support these findings of the study are contained in this article.
